# Swimming in Light: A Large-Scale Computational Analysis of the Metabolism of *Dinoroseobacter shibae*

**DOI:** 10.1371/journal.pcbi.1003224

**Published:** 2013-10-03

**Authors:** Rene Rex, Nelli Bill, Kerstin Schmidt-Hohagen, Dietmar Schomburg

**Affiliations:** 1 Department of Bioinformatics and Biochemistry, Technische Universität Braunschweig, Braunschweig, Germany; The Pennsylvania State University, United States of America

## Abstract

The *Roseobacter* clade is a ubiquitous group of marine *α*-*proteobacteria*. To gain insight into the versatile metabolism of this clade, we took a constraint-based approach and created a genome-scale metabolic model (*i*Dsh827) of *Dinoroseobacter shibae* DFL12T. Our model is the first accounting for the energy demand of motility, the light-driven ATP generation and experimentally determined specific biomass composition. To cover a large variety of environmental conditions, as well as plasmid and single gene knock-out mutants, we simulated 391,560 different physiological states using flux balance analysis. We analyzed our results with regard to energy metabolism, validated them experimentally, and revealed a pronounced metabolic response to the availability of light. Furthermore, we introduced the energy demand of motility as an important parameter in genome-scale metabolic models. The results of our simulations also gave insight into the changing usage of the two degradation routes for dimethylsulfoniopropionate, an abundant compound in the ocean. A side product of dimethylsulfoniopropionate degradation is dimethyl sulfide, which seeds cloud formation and thus enhances the reflection of sunlight. By our exhaustive simulations, we were able to identify single-gene knock-out mutants, which show an increased production of dimethyl sulfide. In addition to the single-gene knock-out simulations we studied the effect of plasmid loss on the metabolism. Moreover, we explored the possible use of a functioning phosphofructokinase for *D. shibae*.

## Author Summary

The oceans are home to a large variety of microorganisms, which interact in several ways with world-wide metabolic cycles. A representative of an important group of marine bacteria called the *Roseobacter* clade is *Dinoroseobacter shibae*. This organism is known to use a variant of photosynthesis to obtain energy from light. Another feature of *D. shibae* and many other *Roseobacters* is the ability to degrade an abundant compound in the ocean called dimethylsulfoniopropionate. Importantly, one degradation pathway of dimethylsulfoniopropionate releases a side product, which affects climate by seeding cloud formation. In this work, we constructed a genome-scale metabolic model of *D. shibae* and carried out a detailed computational analysis of its metabolism. Our model simulates the light-harvesting capabilities of *D. shibae* and also accounts for the energy needed to swim. Thanks to our exhaustive simulations we were able to elucidate the effect of light on the growth of *D. shibae*, to study the consequences of genetic perturbations, and to identify mutants which produce more cloud-seeding compounds. Foremost, our computational results help to understand an important part of the complex processes in the ocean in greater detail. Besides, they can be a valuable guide for future wet-lab experiments.

## Introduction

The *Roseobacter* clade is a versatile group of Gram-negative *α*-*proteobacteria*, which can be found in all oceans worldwide. Especially during phytoplankton blooms they account for a large fraction of the marine bacterial community [1], [2]. Here, we focus on the aerobic anoxygenic phototroph *Dinoroseobacter shibae* DFL12T [3]. Although the bacterium has been isolated from the surface of the dinoflagellate *Prorocentrum lima*, it can also be motile by the means of a single polar flagellum. It harbors five plasmids and needs additional vitamins (biotin, nicotinate, and 4-aminobenzoate) to grow in minimal seawater medium. To obtain energy, *D. shibae* can use oxygen, nitrate or dimethyl sulfoxide (DMSO) as terminal electron acceptor. Additionally, energy generation is possible via light dependent aerobic anoxygenic photosynthesis [3], [4].

Despite of their common taxonomic classification, many members of the *Roseobacter* clade have adopted a unique life style and accordingly have developed a tailored metabolism [1], [5]. For instance, *D. shibae* is believed to live in symbiosis with its host and to provide the algae with vitamin B12 in exchange for carbon sources originating from photosynthesis [6], [4]. Some members of the *Roseobacter* clade produce storage compounds belonging to the group of polyhydroxyalkanoates, which are biopolymers with a potential industrial use [7], [8]. Under optimal conditions, *Dinoroseobacter* sp. JL1447, a close relative of *D. shibae*, has been found to produce large quantities of polyhydroxyalkanoates [9]. Moreover, *D. shibae* and other *Roseobacters* produce dimethyl sulfide (DMS) during DMSO respiration and if dimethylsulfoniopropionate (DMSP) is used as carbon source. This molecule contributes to the characteristic odor of the ocean and affects climate by seeding cloud formation [10]. All these properties demonstrate that *D. shibae* notably differs from well-studied organisms.

For the aforementioned reasons, the *Roseobacter* clade and its members were subjects of intensive research in the past few years [1], [2]. Since the initial description of *D. shibae* in 2005, further studies on the genome sequence and transcriptome analyses under changing illumination conditions have been published [3], [4], [11]. Furthermore, important parts of the metabolism of *D. shibae* were elucidated by ^13^C labeling experiments, a study on DMSP catabolism, and a study targeting energy conservation [12], [13], [14]. Remarkably, no phosphofructokinase activity has been observed in *D. shibae* during growth on glucose [12]. Recently, a basic metabolic model of *Rhodobacter sphaeroides*, another member of the *Roseobacter* clade, has been created [15]. However, no systematic and detailed computational analysis of the metabolism of any member of this ubiquitous group of marine bacteria has been carried out to date.

In this work, a large-scale computational analysis of the metabolism of the marine bacterium *Dinoroseobacter shibae* DFL12T is presented. Prior to the analysis, we created an elaborate genome-scale metabolic model [16], [17] of *D. shibae*, denoted *i*Dsh827. It has been validated against experimental data and covers 827 open reading frames. Moreover, *i*Dsh827 is the first genome-scale metabolic model which explicitly takes the energy demand of bacterial motility into account. Additionally, our model is the first one which uses aerobic anoxygenic photosynthesis. In total, 391,560 distinct simulations featuring a large variety of different growth conditions, e.g. varying carbon and nitrogen sources, aerobic and anaerobic conditions, and the availability of light have been carried out. A large fraction of the simulations is dedicated to plasmid and single gene knock-out mutants. In detail, the aim of this work is to

- analyze metabolic fluxes in *D. shibae* with focus on energy balance
- validate the predictions of the metabolic response to light via metabolome analyses
- introduce the energy demand of motility as important parameter of metabolic models
- provide a possible explanation for aerobic denitrification
- identify conditions enhancing the usage of the DMSP demethylation pathway
- explore the potential role of the phosphofructokinase in *D. shibae*
- study the effect of plasmid loss
- elucidate the behavior of single gene knock-out mutants and
- find mutants producing significantly more cloud-seeding DMS than the wild type.

## Results

### Biomass composition

A fundamental assumption made in most constraint-based simulations is that the organism tries to maximize its growth rate as much as possible under the given environmental conditions. Therefore, the model *i*Dsh827 contains a biomass reaction, which consumes appropriate quantities of 113 different metabolites needed for the reproduction of *D. shibae*. The flux through this reaction corresponds to the growth rate and is the objective function of all simulations presented here. Hence, to obtain precise adjustments, the contribution of each metabolite to the biomass composition was either quantified experimentally for *D. shibae* or estimated based on literature values of related organisms (Table 1). For a detailed description of the experimental procedures used to determine the different fractions see the materials and methods section.

**Table 1:** Biomass composition used in the model *i*Dsh827.

Component	Percentage	Reference	Organism
Protein	45.34%	this work	*D. shibae*
DNA	3.22%	this work	*D. shibae*
RNA	4.11%	this work	*D. shibae*
Lipids	14.02%	this work	*D. shibae*
Polyhydroxybutyrate	24.70%	this work	*D. shibae*
Bacteriochlorophyll *α*	0.08%	[3]	*D. shibae*
Lipopolysaccharides	4.00%	[20]	*R. denitrificans*
Peptidoglycan	2.50%	[18]	*E. coli*
Components with unknown fraction	0.10%	estimated	
Soluble pool	1.91%	remainder	

Literature values originating from *Roseobacter denitrificans* were used to estimate the amount of lipopolysaccharides produced by *D. shibae*. Furthermore, the fraction of peptidoglycan was presumed to be approximately equal to the values determined for *Escherichia coli* [18]. Moreover, compounds whose proportion of the biomass is still unknown were estimated to make up 0.1% of the total dry weight all together. Finally, the soluble pool was assumed to account for the remaining biomass fraction. Since some biomass compounds could not be separated by the analytical procedures used during the laboratory experiments, we corrected the measured values for these fractions. As the original lipid fraction also contained the lipopolysaccharide and the bacteriochlorophyll *α* fractions, we subtracted these values to obtain the actual lipid content. Moreover, we corrected the original protein fraction by subtracting the peptidoglycan value. The relative portions of the nucleotides and amino acids were estimated from the genome sequence as suggested by the reconstruction protocol [19]. The predominant respiratory lipoquinone (ubiquinone-10) and the predominant cellular fatty acid (18:1*ω*7*c*) were chosen to represent their corresponding compound group [3]. Furthermore, the ratio of Kdo2-lipid A to the O-antigen in the lipopolysaccharide was calculated from values measured for *Roseobacter denitrificans* [20]. Under anaerobic conditions, the oxygen atoms in the singlet oxygen quencher spheroidenone neither originate from water nor from CO_2_ but probably from other cell components [21]. Hence, we included its precursor methoxyneurosporene in the biomass reaction. A detailed measurement of the content of the soluble pool is only available for *E. coli* [22]. Nevertheless, we used these values to constitute the soluble pool content in *i*Dsh827. Therefore, we removed compounds which were either specific for the growth conditions used in the study or which were not part of our model. As the genome annotation did not give any indications for spermidine production in *D. shibae*, we did not include this compound into the biomass reaction. Due to the fact that aerobic anoxygenic phototrophs produce bacteriochlorophyll *α* exclusively in the dark under aerobic conditions [23], we included two biomass reactions in the model: one with and one without bacteriochlorophyll *α*. The former reaction contains a term corresponding to 4 *nmol/mg_protein_* as determined for *D. shibae* [3]. Before running a simulation, the appropriate biomass reaction is enabled automatically based on the preset conditions. Both biomass reactions are normalized to one gram dry weight.

### Nutrient sources used by *D. shibae*

To elucidate the usability of carbon sources by *D. shibae*, we conducted Phenotype MicroArray experiments. Respiration occurred on the carbonic acids succinate, fumarate, 2-oxoglutarate, L-lactate, acetate, propionate, (R)-3-hydroxybutanoate, glycolate, glyoxylate, pyruvate, the carbohydrates *α*-D-glucose, D-fructose, L-rhamnose, maltose, D-xylose and on the sugar alcohols D-ribitol, myo-inositol, D-arabitol, and D-xylitol. Out of 190 different carbon sources tested, *D. shibae* was able to utilize only 19, which confirms the poor variety of potential nutrients used by the organism [3]. Most of the mentioned carbon sources were applied for growth experiments in batch cultures and gave a positive biomass yield. The maximal growth rate on the reference substrate succinate was 0.25 h^−1^, on glyoxylate 0.15 h^−1^.

### Distribution of swimming velocities

**Figure 1:**
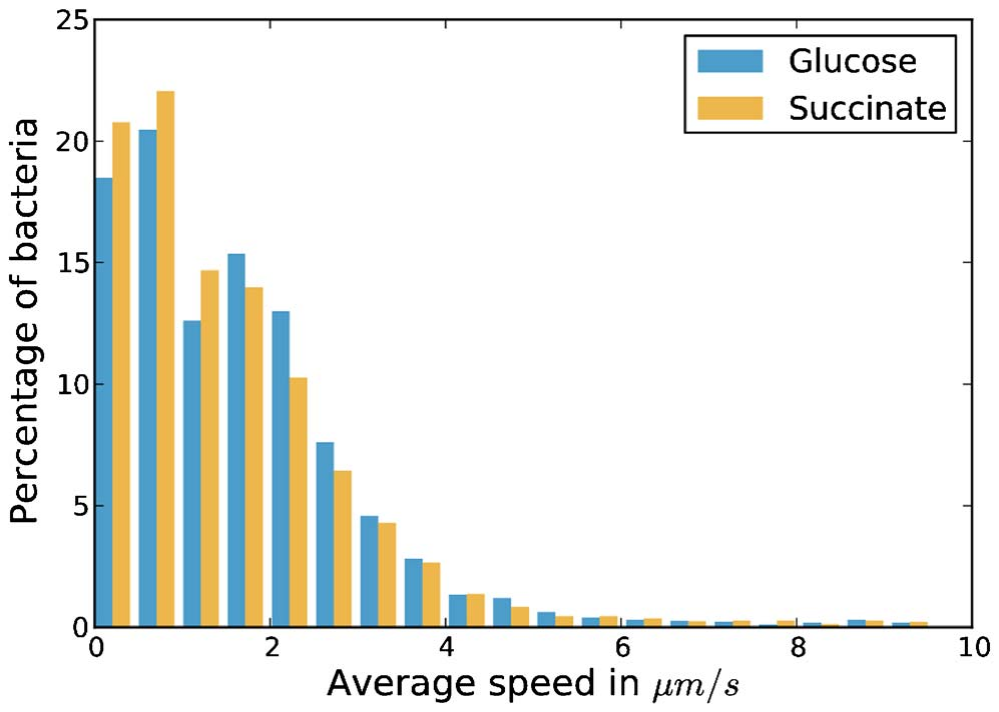
Histogram of average swimming velocities of *D. shibae*. The velocities are given in *µm/s* and the heights of the bars indicate the fraction of bacteria displaying this velocity.

As not all cells in a bacterial culture display the same degree of motility [24], we tracked the movement of *D. shibae* cells grown on glucose and succinate experimentally to determine the distribution of swimming velocities. The resulting frequency distributions, shown in Figure 1, slightly differ from each other. The average velocity of cells is 5% higher (1.68 *µm/s*) on glucose than on succinate (1.59 *µm/s*). However, the shapes of the distributions differ significantly as a two-sample Kolmogorov-Smirnov test yielded a p-value of 1.06×10^−5^.

### *i*Dsh827: A genome-scale metabolic model of *D. shibae*

The reconstructed metabolic network of *D. shibae* gives insight into the metabolism of this representative of the cosmopolitan *Roseobacter* clade. The metabolic model *i*Dsh827 consists of 1488 reactions covering 827 open reading frames (Table 2) and is based upon an aggregated genome annotation provided by the EnzymeDetector database [25]. To fill gaps in important pathways, we included a few enzymes with low evidence scores into the model. A graphical representation of the distribution of evidence scores can be seen in Supplementary Figure S1 and the complete list of all genes covered in *i*Dsh827 is given in Supplementary Dataset S1. Moreover, 199 genes retrieved from the annotation database were not taken into consideration either because their gene product was a non-metabolic enzyme or because the annotation was ambiguous.

**Table 2:** Properties of the genome-scale metabolic model *i*Dsh827.

Property	Value	
Total number of genes in *D. shibae*	4,245	
Genes covered	827	(19.4%)
Enzymes	622	
Reactions	1488	
Non-blocked reactions	778	
Distinct biomass compounds	113	

To reproduce growth on minimal medium as accurately as possible, multiple refinements guided by experimental results found in the literature were made. Important refinements are highlighted in the next sections. The final metabolic model in the SBML format [26] can be found in Supplementary Dataset S2 and in the BioModels database (accession ID: MODEL1308180000).

#### Manually added or modified reactions

To fill gaps in metabolic pathways essential for the synthesis of biomass components, some reactions were added manually. These reactions have either no EC number assigned, contain generic metabolites or are stoichiometrically unbalanced. For instance, some enzymes of the thiamin synthesis pathway lack EC numbers [27]. Furthermore, the phosphatidylglycerol synthesis was constructed to use fatty acids with correct lengths. In some cases, generic cofactors had to be replaced by actual metabolites as found in the BRENDA [28]. To allow the anaerobic synthesis of ubiquinol, we added hydroxylation reactions in which the molecular oxygen is replaced by water [29]. The aerobic anoxygenic photosynthesis was modeled by two reactions: One reaction consumes the photons at the reaction center and the other one translocates two protons via cytochrome bc1 [30]. As the phosphofructokinase is known to be inactive in *D. shibae*, we constrained the flux through the corresponding reactions to zero [12].

#### Transport and boundary reactions

Tripartite ATP-independent periplasmic transporters predominate in the genome of *D. shibae* [4]. Hence, we included sodium symporters for each carbon source in the model. As a notable exception, we allowed glycerol and glyoxylate to diffuse freely through the membrane using channel proteins. Otherwise, growth on these carbon sources could not be reproduced by the model, although growth was experimentally verified. In order to achieve comparable results, we normalized the uptake of the carbon sources to the number of carbon atoms. Hence, for a given uptake rate, the number of carbon atoms taken up per time is the same for all carbon sources. The medium (12 carbon atoms *mmol/*(*gDW h*) and the high (39.2 carbon atoms *mmol/*(*gDW h*) uptake rates correspond to the experimentally determined uptake rate of glucose and succinate, respectively. Additionally, we simulated nutrient-poor conditions by limiting the uptake rate to only one carbon atom per gram dry weight and hour. Furthermore, we included a sodium/proton antiporter, which exchanges two protons for one sodium ion [31], [32]. Thus, the sodium ions imported together with the carbon source are able to leave the cell. The number of protons transported over the membrane in the reactions of the oxidative phosphorylation correspond to the values determined for *E. coli* [18], [33]. A list of all proton-translocating reactions is given in Table 3.

**Table 3:** Proton-translocating reactions in *i*Dsh827.

Reaction or Enzyme name	Reaction equation
**cytochrome-c oxidase**	8 H + + 1 oxygen + 4 Cytochrome-C-Reduced→2 H2O + 4 Cytochrome-C-Oxidized +4 H +*_ex_*
**NADH:ubiquinone reductase (H+-translocating)**	1 NADH + 5 H + + 1 Ubiquinone↔1 NAD + + 1 Ubiquinol + 4 H +*_ex_*
**ubiquinol oxidase**	1 oxygen + 4 H + + 2 Ubiquinol→4 H +*_ex_*+ 2 H2O + 2 Ubiquinone
**H+-transporting two-sector ATPase**	1 ATP + 3 H + + 1 H2O↔1 ADP + 1 phosphate + 4 H +*_ex_*
**ubiquinol-cytochrome-c reductase**	1 Ubiquinol + 2 Cytochrome-C2-Oxidized + 2 H+→1 Ubiquinone + 2 Cytochrome-C2-Reduced + 4 H + *_ex_*
**Na+/proton antiporter**	1 Na + + 2 H+*_ex_*→1 Na +*_ex_*+ 2 H +
**flagellar motor proton flux**	1 H +*_ex_*→1 H +

In addition to the transporters and boundary reactions for the different nutrients, we included reactions which supply the model with the vitamins biotin, nicotinate, and 4-aminobenzoate essential for growth of *D. shibae* [3]. Moreover, we incorporated the usage of light as an energy source by introducing a photon boundary reaction fixed to an uptake rate of 33.8 photons *mmol/*(*gDW h*). We fitted this value to reproduce the decrease of the oxygen uptake of *D. shibae* when exposed to light [14]. Another uncommon boundary reaction exclusively supplies oxygen for the production of the vitamin B_12_ precursor *α*-ribazole. This is necessary because no anaerobic alternative for the reaction catalyzed by the 5,6-dimethylbenzimidazole synthase is currently known. Aside from boundary reactions for the volatile products usually excreted by microorganisms (including DMS and methanethiol in the case of *Roseobacters*), we added additional sink reactions to allow compounds which cannot be degraded to leave the system. For instance, the 5,6-dimethylbenzimidazole synthase already mentioned above produces an unknown compound (possibly dialuric acid [34]), which is disposed by a boundary reaction. Furthermore, 5′-deoxyadenosine is created during the synthesis of thiamin. In our model, the adenine is salvaged [35] and the remaining 5-deoxy-D-ribose is excreted. Another side product of the thiamin synthesis is 4-methylphenol, which may accumulate in the cell [36]. Moreover, glycolaldehyde is created by the dihydroneopterin aldolase eventually leading to tetrahydrofolate. Although glycolaldehyde is predicted to be degraded by a low-specificity L-threonine aldolase, in some simulations a sink reaction was necessary. The total fraction of carbon lost through these additional sink reactions is four orders of magnitude smaller than the carbon input flux and thus has little impact on our results. Finally, secretion reactions for lactate, acetate, urea, urate, and L-glutamine were included. However, our simulations revealed that none of these boundary reactions have to be active in any of the wild type physiological states considered here.

#### Energy demand of motility

The bacterial flagellar motor in *D. shibae* is homologous to the machinery in *E. coli* and hence it is likely driven by the proton-motive force [37]. Remarkably, the energy spent on mobility is negligible in enteric bacteria but marine bacteria are much more mobile [38], [39]. Hence, we incorporated this energy demand using a reaction, which transfers a certain amount of protons from the periplasmic space to the cytosol. The actual value depends on the fraction of motile bacteria chosen for the specific simulation run (see Table 4).

**Table 4:** Conditions whose combinations were covered in the simulations.

Parameter	Cardinality	Possible values
Carbon source	26	(R)-3-hydroxybutanoate, (R)-lactate, (S)-lactate, 2-oxoglutarate, acetate, alpha-D-glucose, alpha-D-xylopyranose, alpha-L-rhamnose, alpha-maltose, beta-D-fructofuranose, *β*-D-glucose, citrate, D-ribitol, D-xylitol, DMSP, ethanol, fumarate, glycerol, glycolate, glyoxylate, L-glutamate, myo-inositol, polyhydroxybutyrate, propionate, pyruvate, succinate
Nitrogen source	3	ammonia, urea, nitrate
Sulfur source	1	sulfate
Phosphorus source	1	phosphate
Terminal electron acceptor	5	oxygen, nitrate, dimethyl sulfoxide, oxygen and nitrate, oxygen and dimethyl sulfoxide
Carbon uptake rate	3	1 (low), 12 (medium), 39.2 (high)
Illumination	2	light (34.6 photons *mmol/*(*gDW h*)), dark (0 photons *mmol/*(*gDW h*))
Fraction of motile bacteria	3	0%, 10%, 20%

The uptake rate of the carbon source is given in *mmol* carbon atoms per *gram* dry weight and *hour*.

### Large-scale computational analysis

**Figure 2:**
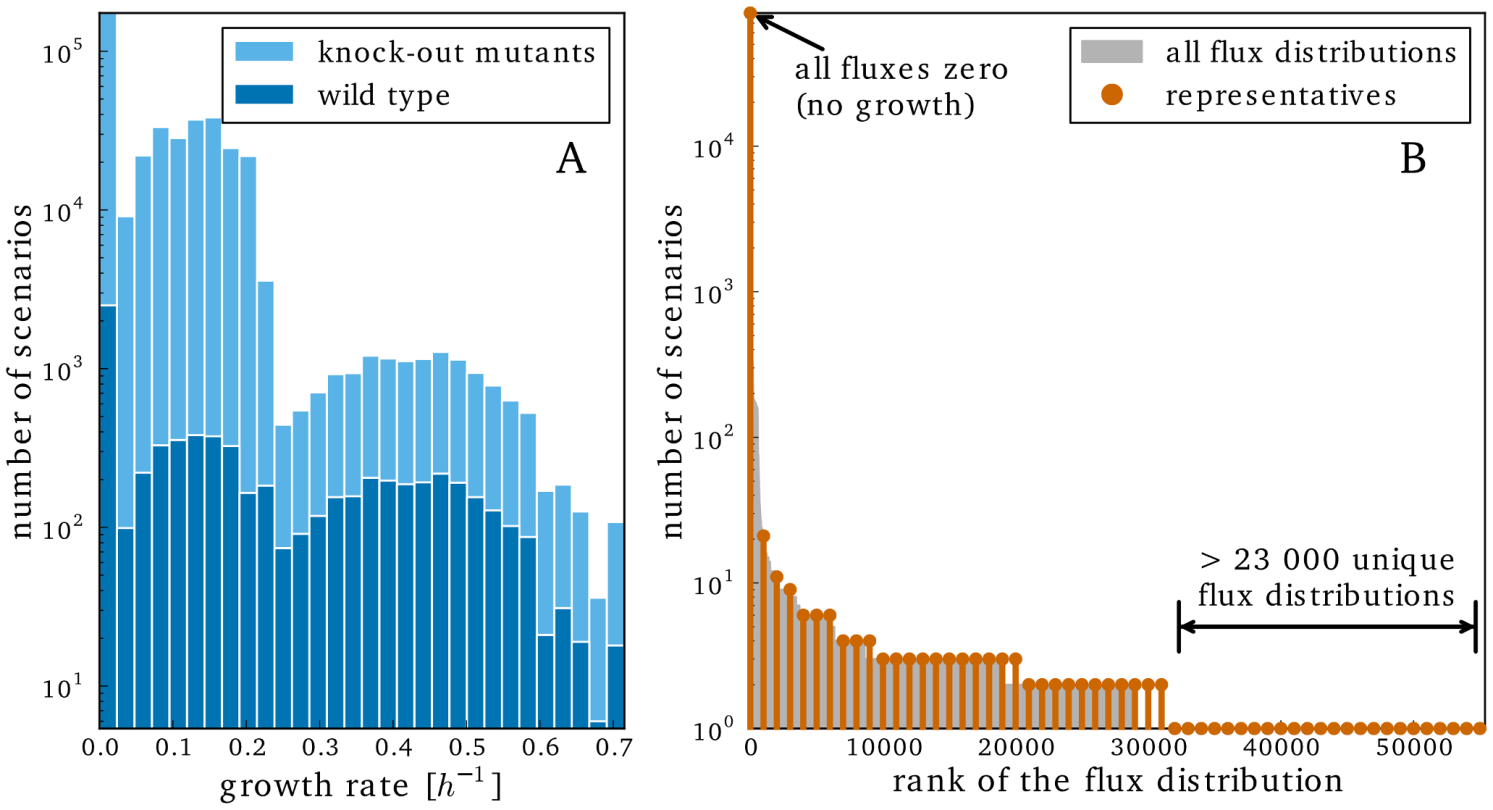
High-level overview of all simulations. A) Histogram showing the distribution of growth rates over all simulations. B) Number of simulations displaying a given flux distribution ranked by the number of simulations. For clarity, every 1000th flux distribution was chosen as representative.

In total, we carried out 391,560 simulations to study the metabolic network of *D. shibae* under various environmental conditions (Table 4) and the effect of genetic perturbations in terms of single gene and plasmid knock-outs. Figure 2A gives a rough summary of all simulations. The leftmost bar corresponds to physiological states with no or only very little growth. Furthermore, the modes visible in the figure are mainly caused by the different carbon uptake rates and other environmental conditions.

As visualized in Figure 2B, some simulations of distinct states yielded exactly the same flux distribution. This happens if the changed parameter has no effect on the metabolism. For instance, an additional nutrient may remain unused or the function of a knocked-out gene can be carried out by another one. Hence, we observed only 55,390 distinct flux distributions in our simulations (Figure 2B). Prominently, the most common distribution is the one where no fluxes are active at all and hence no growth occurs. This is often due to insufficient carbon uptake or a lethal knock-out. In contrast, more than 23,000 flux distributions are unique for only one physiological state. In the following sections, we will take a closer look at the fluxes in some selected simulations.

#### Growth of the wild type under various conditions

**Figure 3:**
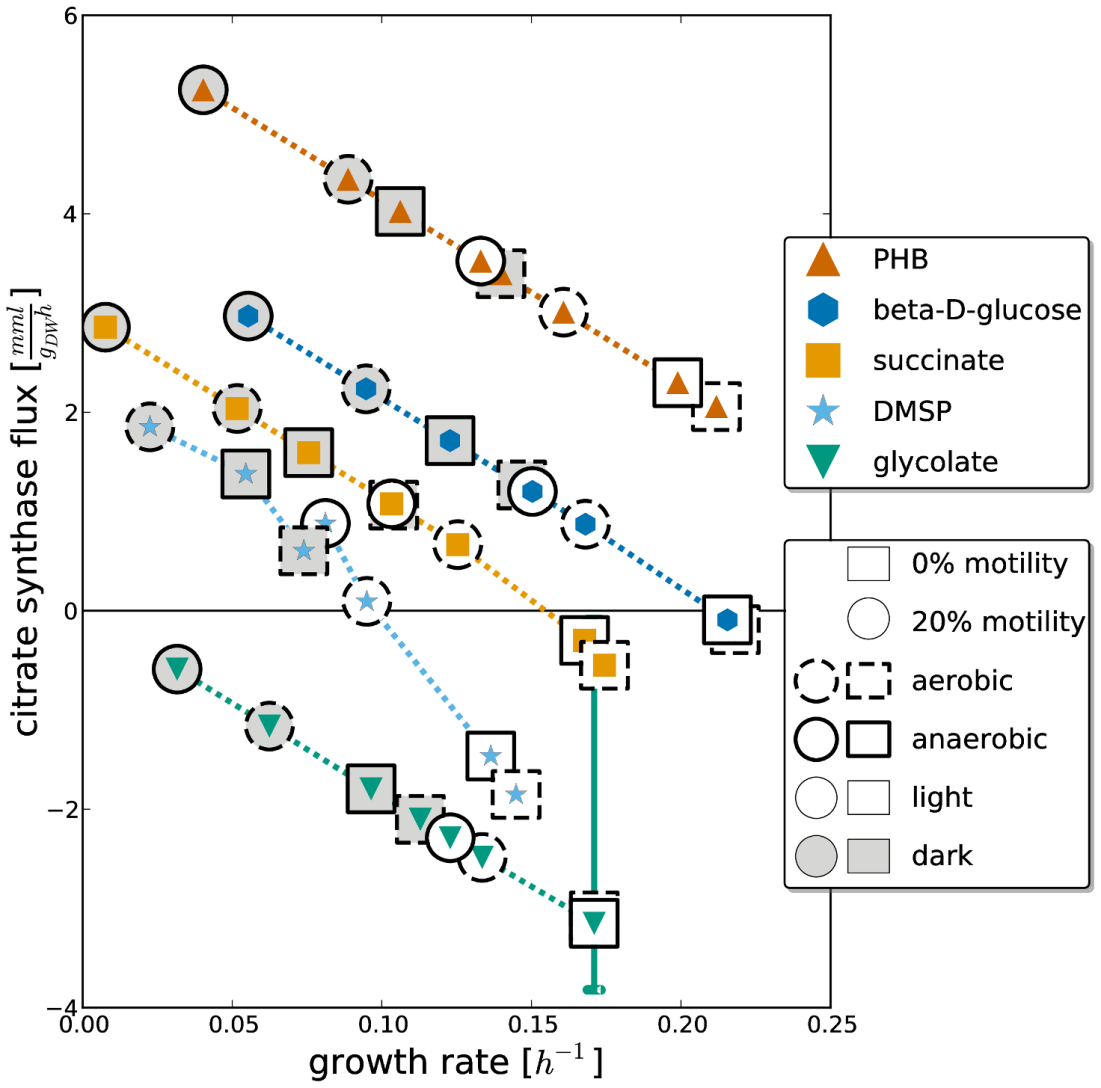
Overview of the wild type simulations. In this figure the growth rate is charted against the flux through the citrate synthase. The carbon source is given by the color and shape of the inner marker. Moreover, the outer marker indicates motility (shape), aerobic/anaerobic conditions (line style), and illumination (background color). No growth was predicted in any high motility physiological state for dark and anaerobic conditions if DMSP was used as carbon source. Hence, this entry is missing in the figure. PHB: polyhydroxybutyrate; DMSP: dimethylsulfoniopropionate.

The first set of 7,020 simulations, was carried out to study the behavior of the wild type strain under different environmental conditions. Therefore, we varied one condition at a time and ran a simulation for each possible combination of parameters (see Table 4). In addition to the different nutrient sources, we also varied the uptake rate of the carbon source, the availability of terminal electron acceptors, the illumination, and the fraction of motile bacteria. The set of all carbon sources used in the simulations consists of the carbon sources determined by Biebl et al. [3], the usable carbon sources identified in our Phenotype MicroArray experiments, and additionally polyhydroxybutanoate, representing the storage compound of *D. shibae*. As *D. shibae* is able to grow aerobically and anaerobically, we simulated physiological states with oxygen, DMSO or nitrate as terminal electron acceptor or a combination of DMSO or nitrate with oxygen. A condensed overview of the wild type simulations can be seen in Figure 3. While the x-axis represents the growth rate, the y-axis depicts the flux through the citrate synthase (positive values correspond to the formation of citrate). The lines, which resemble error bars, indicate the minimal and maximal flux through the reaction possible during a simulation determined by flux variability analysis. Hence, we term them *variance bars*. For the sake of clarity, this figure is limited to five carbon sources, the medium uptake rate, and ammonia as nitrogen source. For each carbon source, eight physiological states with different terminal electron acceptors (oxygen/DMSO), illumination conditions (dark/light), and motility fractions (no or 20% motility) are shown. The nitrate simulations are not shown in this figure because they coincide with the DMSO simulations.

In general, we observe that oxygen supports faster growth than DMSO and that light significantly enhances the growth on all carbon sources. Moreover, simulations with a high motility rate display a strongly impaired growth rate. In all dark states, except for the glycolate states, the citrate synthase runs in forward direction. Under light conditions, the activity of the citrate synthase is considerably reduced or even reversed. Notably, the anaerobic glycolate simulation in light is the only one which produces visible variance bars.

**Figure 4:**
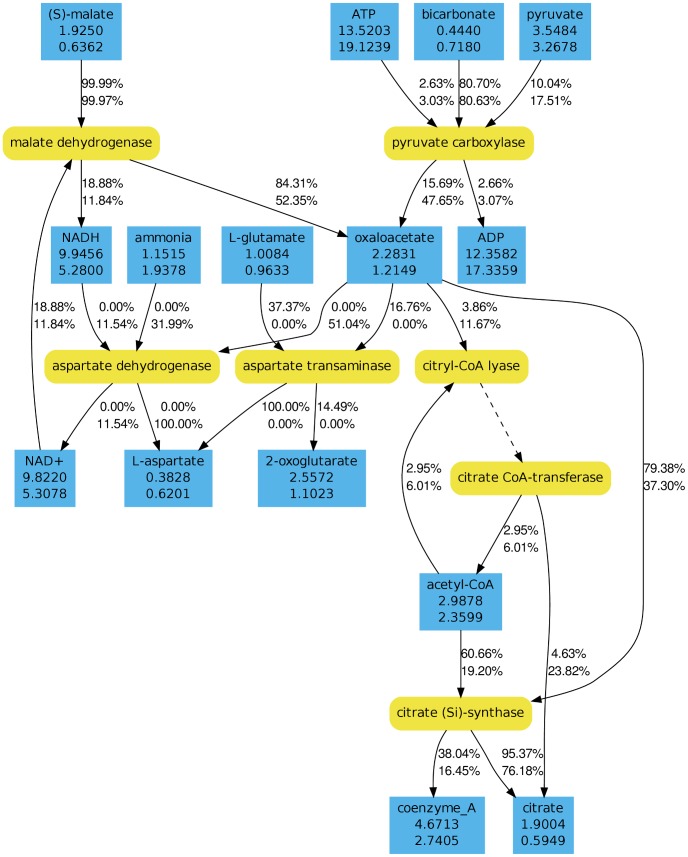
Metabolic context of oxaloacetate during growth on glucose. Reactions are shown in yellow (round boxes) while metabolites are shown in blue (angular boxes). The total flux through a metabolite is given in *mmol/*(*g_DW_ h*). Outgoing and incoming fluxes are given in percentages of the total flux. The upper numbers correspond to a dark states and the lower numbers to light states. In both cases growth on glucose was simulated under aerobic conditions.

**Figure 5:**
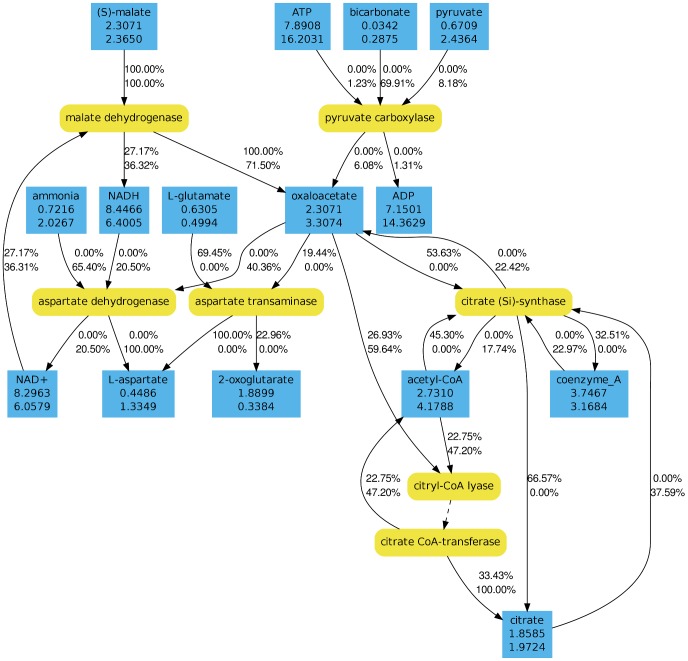
Metabolic context of oxaloacetate during growth on DMSP. Reactions are shown in yellow (round boxes) while metabolites are shown in blue (angular boxes). The total flux through a metabolite is given in *mmol/*(*g_DW_ h*). Outgoing and incoming fluxes are given in percentages of the total flux. The upper numbers correspond to a dark states and the lower numbers to light states. In both cases growth on DMSP was simulated under aerobic conditions.

In order to get a more detailed view on the effect of light on the metabolism, we analyzed the flux distributions near the metabolic branch point oxaloacetate using the software AMEBA [40]. Given one or more nodes of the metabolic network AMEBA allows to visualize the metabolic context and the corresponding flux distributions. The analysis revealed strong flux changes in response to light. Details for the carbon sources glucose and DMSP can be seen in Figures 4 and 5, respectively. On both carbon sources the relative contribution of the anapleurotic synthesis of oxaloacetate by the pyruvate carboxylase increases in light. However, the effect is much more pronounced on glucose because on DMSP oxaloacetate is also created by the citrate synthase operating backwards. As consequence, the total flux through oxaloacetate in light decreases on glucose but increases on DMSP. Similar effects apply to the carbon source succinate. Metabolome analyses showed a 1.7-fold increase of citrate in light indicating an accumulation due to a reduced TCA cycle activity. Furthermore, our simulations predicted a decrease of the carbon dioxide excretion rate by 19.6% in light on succinate, which is quite close to the experimentally determined specific carbon dioxide production rate decrease of 12% (data not shown). As already indicated by our results above, the flux through the TCA cycle is reduced or even reversed in light. Hence, an increased amount of oxaloacetate is routed to anabolic pathways.

#### Aerobic denitrification in light

When applying a physiological state where oxygen and nitrate are available, our simulations predict that parallel usage of both electron acceptors is possible under certain conditions, independently from the nitrogen source. However, this occurs only in light and on the carbon sources 2-oxoglutarate, acetate, (R)-3-hydroxybutanoate, L-glutamate, polyhydroxybutyrate, DMSP, myo-inositol, and ethanol. The effect is restricted to the lowest carbon uptake rate for most of these carbon sources. However, L-glutamate supports aerobic denitrification also with the medium uptake rate and ethanol displays the described behavior for all uptake rates covered in the simulations.

#### Usage of DMSP degradation pathways

**Figure 6:**
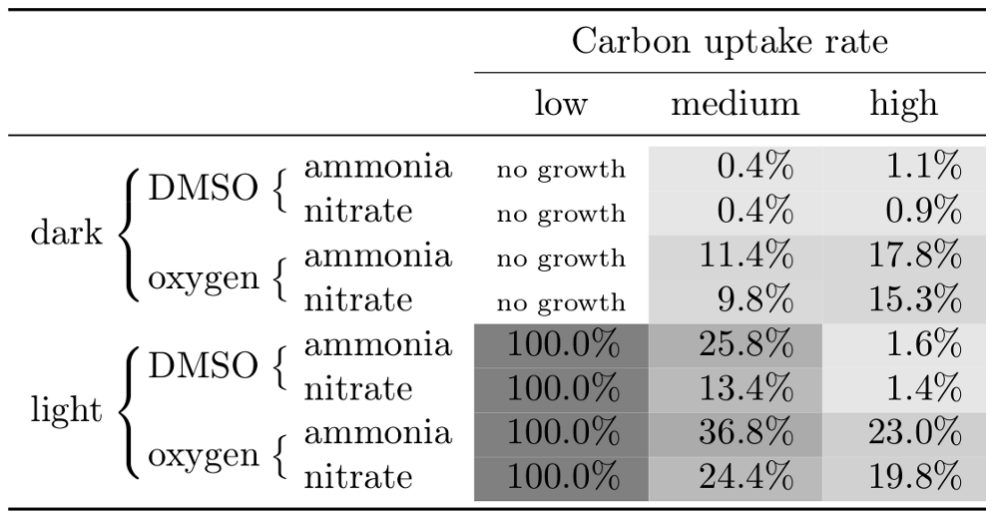
Relative contribution of the DMSP demethylation pathway in dependence of varying simulation parameters. The percentage and the background color indicate the maximal fraction of demethylated DMSP determined by flux variability analysis while the remainder represents the DMSP degraded by the cleavage pathway. The maximal difference to the minimal contribution is 1.2%.

In the genome of *D. shibae*, three genes coding for different DMSP-degrading enzymes are present [41], [10]. One gene product (DmdA, Dshi_2320) catalyzes the demethylation of DMSP while the other two (DddL, Dshi_3313 and DddD, Dshi_3632) cleave DMSP. All three DMSP degradation pathways produce volatiles that are excreted to the environment. The demethylation pathway produces methanethiol whereas the cleavage pathways produce DMS. From a stoichiometric point of view, the cleavage pathways are equivalent. Hence, we focused our analysis on the relative contribution of the demethylation pathway compared to the cleavage pathways. In Figure 6 the contribution of the demethylation pathway under diverse conditions in dependence of the carbon uptake rate is shown. These values represent the maximal fraction of DMSP degraded by the gene product of DmdA, as determined by flux variability analysis. If the carbon uptake rate is low, DMSP supports growth only in presence of light by using the demethylation pathway exclusively. In darkness, the available carbon is not sufficient to cover the basic energy demand of the cell – namely the non-growth-associated maintenance requirement and the proton flux for motility. Furthermore, we observed an increased usage of the demethylation pathway for the medium uptake rate in light compared to the dark. Interestingly, the values of the high uptake rate do not follow the same pattern. In these cases, more DMSP is demethylated under aerobic than under anaerobic conditions. To a lesser extend this is also true for the medium uptake rate. Moreover, the usage of nitrate as a nitrogen source correlates with a decreased usage of the demethylation pathway compared to ammonia.

#### Enabling the phosphofructokinase gene

**Figure 7:**
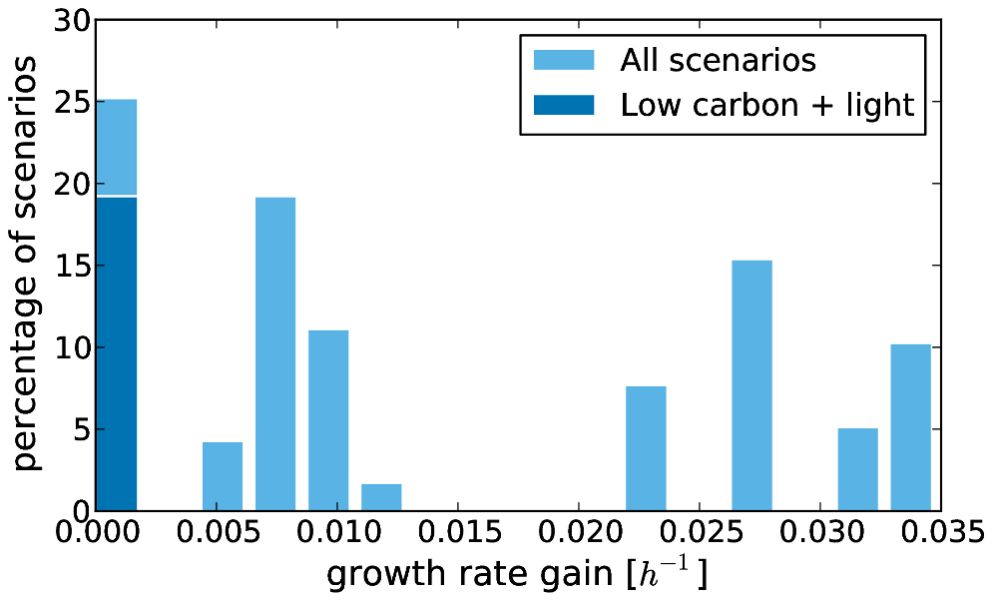
Histogram of the growth rate gain observed if phosphofructokinase is enabled in all simulations with glucose as carbon source. The simulations representing oligotrophic states in light are shown in dark blue. Simulations in which both strains do not display growth are not considered.

To study the effect of a functional phosphofructokinase, we carried out the same set of simulations for a phosphofructokinase enabled mutant as described above for the wild type. In agreement with established biochemical knowledge, only physiological states with glucose as carbon source were affected by this modification. While the maximal increase of the growth rate is 0.035 *h* ^−1^, the mean increase is only 0.014 *h* ^−1^ (Figure 7). However, not all glucose states displayed enhanced growth (leftmost bar). More specifically, none of the illuminated states with a low carbon uptake rate has an increased growth rate (dark blue bar).

#### Effect of plasmid loss

Another set of 43,740 simulations explored the effect of a loss of all or a single plasmid using the same simulation parameters as mentioned above. These simulations revealed that the loss of all plasmids or the single loss of the 86 kb plasmid is lethal in all physiological states (but see below). The single loss of each other plasmid is predicted not to be lethal. However, closer inspection revealed that if the 153 kb plasmid is removed, the growth rate is slightly reduced compared to the wild type in a subset of the simulated physiological states. All of those are illuminated aerobic states. However, the phenomenon is restricted to the carbon sources 2-oxoglutarate, L-glutamate and glycerol.

#### Single gene knock-out mutants

**Figure 8:**
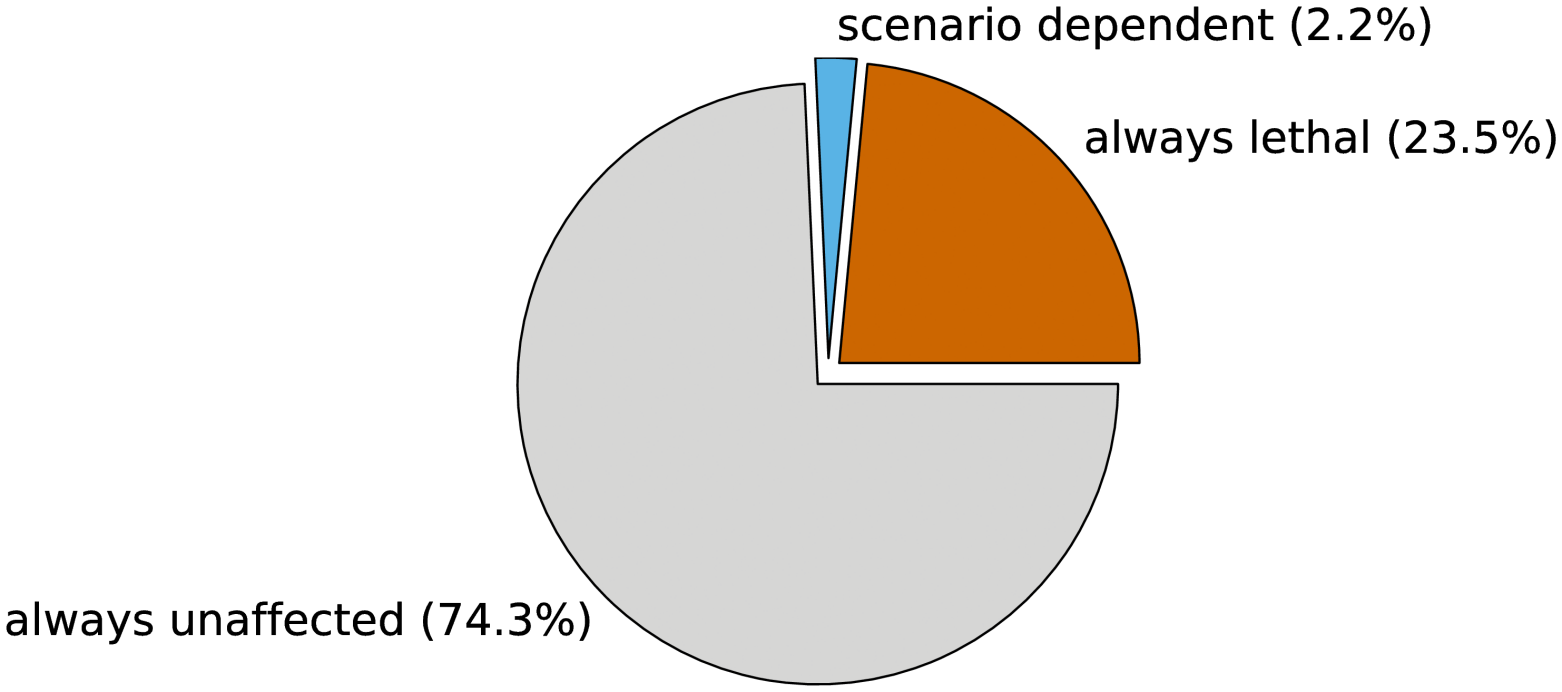
Distribution of the growth phenotype of all 348,300 single gene knock-out mutants.

We studied the effect of single gene knock-outs in 348,300 simulations. As only a loss of all copies of a gene would alter the topology of the metabolic network, we restricted the analyses to genes without paralogous genes. Furthermore, we only simulated physiological states with a medium uptake rate and 10% motile bacteria. All other parameters were varied as shown in Table 4. In most cases (74.3%, see Figure 8) the knock-out did not affect growth in any physiological state. This number includes all genes with paralogous genes. Moreover, 23.5% of all simulated knock-outs were lethal in every physiological state considered here.

**Figure 9:**
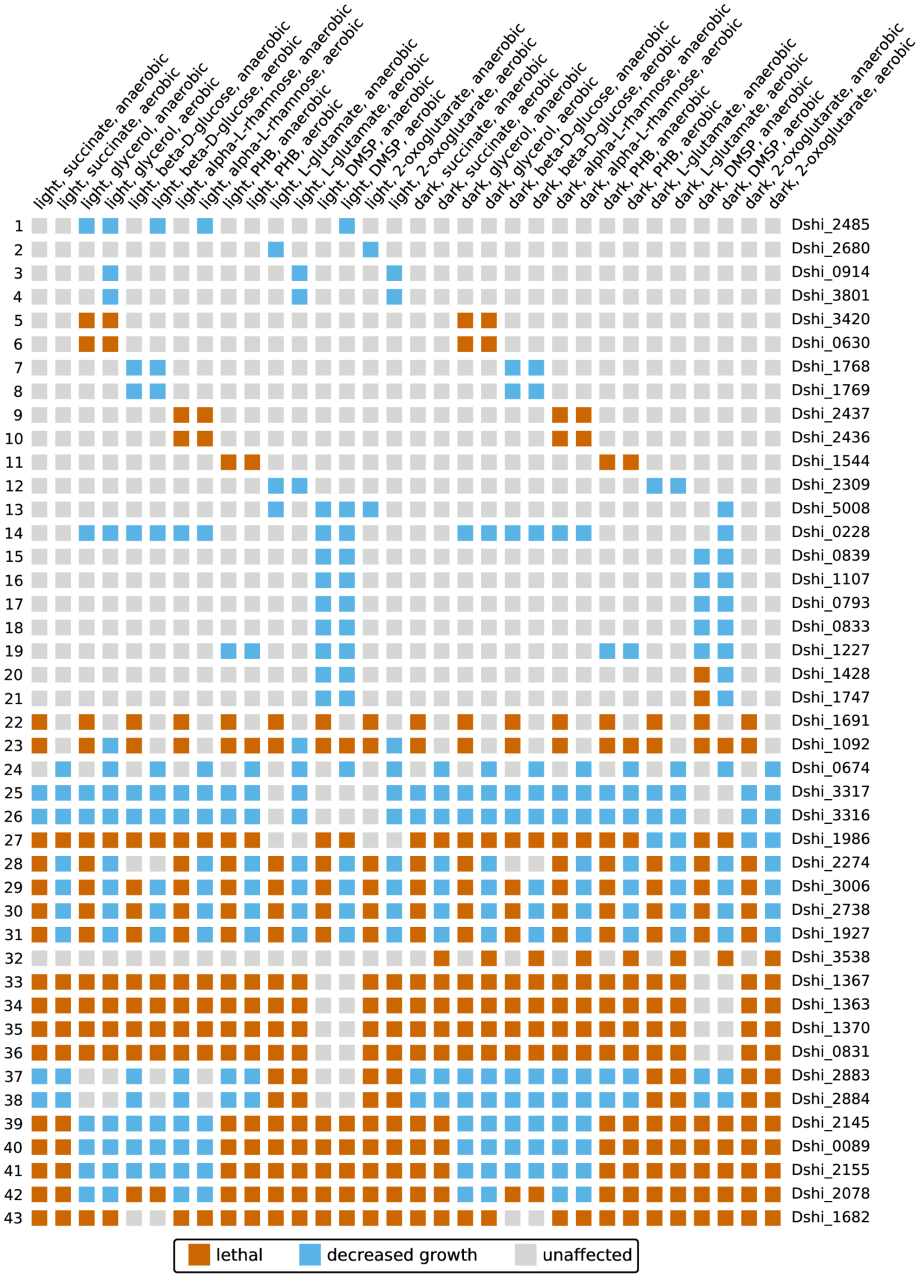
Knock-out matrix showing the growth phenotype of 43 single gene knock-out mutants in selected simulations. Mutants are identified by the locus tag of the disabled gene and physiological states are given by their conditions (illumination, carbon source, and electron acceptor).

Only 63 knock-out mutants (2.2%) displayed a more multifaceted behavior. Details of a selected subset can be seen in the knock-out matrix shown in Figure 9. A complete analysis of each mutant is beyond the scope of this work and certainly requires experimental validation. However, it has not escaped our notice that most mutants show pronounced patterns, which correspond to similar physiological states conditions and thus give insight into the role of the gene in the metabolism. For instance, the cobalt-precorrin-5B (C1)-methyltransferase encoded on Dshi_1691 is needed for the anaerobic synthesis of vitamin B_12_ (Figure 9, line 22). Hence, the deletion of this gene is lethal in all anaerobic states. Moreover, a couple of genes show patterns in dependence of the carbon source (lines five to twelve). The only gene in this set, which lies on a plasmid is Dshi_3801 from the 153 kb plasmid (Figure 9, fourth line). As mentioned above, this gene codes for a catalase. If it is knocked out, the aerobic 2-oxoglutarate, L-glutamate and glycerol simulations in light showed decreased growth. This is consistent with our observations of the plasmid knock-out simulations.

#### Screening for elevated DMS production

A recent simulation studying the effect on climate of doubling DMS emissions [42] inspired us to screen the single knock-out mutants for elevated DMS production. The DMS created from DMSO by the dimethylsulfoxide reductase was not considered in this screening because only an overall increase of cloud-seeding volatiles would affect climate. Thus, we assured that all DMS originates from DMSP. As one might expect, the simulated mutants with a blocked demethylation pathway showed the maximal increase of DMS production compared to the wild type. In aerobic illuminated states their production rate was increased by 58.2% while the growth rate was reduced to 72.7%. Moreover, the L-serine ammonia-lyase mutant (Dshi_5008) also showed considerable values. The DMS production of this mutant was increased by 28.3% and the growth rate decreased to 74.3%. On all other tested carbon sources, growth was not affected.

## Discussion

### Motility is an important model parameter

Although the precise connection between swimming velocity and the corresponding energy demand depends on many unknown variables (e.g. viscosity of the medium and efficiency of the motor protein), it is very likely that the energy demand increases with velocity. As this demand has a great influence on the energy metabolism and hence on growth, this parameter is worth to be considered in genome-scale metabolic models. We suggest to evaluate values within physiological reasonable ranges in multiple simulations to account for the different swimming velocities occurring within a culture and under different environmental conditions.

### Need for efficient transporters

Our simulations showed that the phenotype of *D. shibae* growing on glycerol or glyoxylate can only be reproduced if these compounds are allowed to pass the membrane without active transport. Otherwise, the total energy requirement of the cell, including the energy needed for the active transport, cannot be covered by the degradation of these compounds. Hence, we suppose that other transporters requiring less energy exist in *D. shibae*. Indeed, the existence of a glycerol-conducting channel in *E. coli* is known [43]. Glycerate is already in a highly oxidized state so that the oxidation to CO_2_ does not yield enough energy to sustain growth if the import is ATP-dependent.

### Energy metabolism

In most physiological states shown in Figure 3, the TCA cycle operates in forward direction and thus is used to obtain energy. However, in the simulations with glycolate as sole carbon source the isocitrate lyase converts glyoxylate to D-threo-isocitrate. At this point the flux splits: 80% follow the TCA cycle in forward direction and 20% are routed to citrate. Hence, the citrate synthase runs backwards in the glycolate simulations.

According to our results, the activity of the TCA cycle and the oxidative phosphorylation in general is significantly reduced in light. This is supported by our simulations, the experimentally observed decrease of the specific carbon dioxide production rates, and metabolome analyses during light shift experiments in continuous cultivations of *D. shibae*. Hence, we conclude that the aerobic anoxygenic photosynthesis satisfies the energy demand of the organism in large parts.

Moreover, under illuminated conditions the production of oxaloacetate is increased to supply anabolic reactions. Another effect of the reduced TCA cycle activity is a decreased production of NADPH by the isocitrate dehydrogenase. Hence, the organism must increase the flux through other reactions for compensation under illuminated conditions. For instance, our simulations showed an increased usage of the pentose phosphate pathway in comparison to dark conditions if glucose is used as carbon source. In general, a flexible energy metabolism is probably very important for *D. shibae* because the amount of energy generated by the aerobic anoxygenic photosynthesis can vary greatly. This can be due to changing illumination conditions but also due to the degradation of the light harvesting complex in the course of time [44]. Recent experimental results suggest, that the activity of the aerobic anoxygenic photosynthesis is reduced or even stopped under anaerobic conditions [45]. If this holds for *D. shibae* and all environmental conditions, the illuminated anaerobic simulations would coincident with the dark anaerobic simulations.

As we pointed out in the results section, the anaerobic glycolate simulation in light is the only one displaying variance. This is probably due to an energy overflow, which occurs if more energy (external protons) is generated by the aerobic anoxygenic photosynthesis than needed for growth. Thus, energy-wasting futile reactions can take place in any part of the metabolic network. Futile reactions waste energy and produce heat and thus are usually disadvantageous for the organism. Hence, they are probably tightly regulated. However, the uncertainty stems from the fact that the simulations cannot predict which set of futile reactions take place. A possibility we did not test in our simulations is a leakage of protons through the membrane. Another uncertainty in energy balance is the precise number of periplasmic protons used for motility. This number may vary greatly depending on environmental conditions and the average velocity of the motile bacteria [38].

Furthermore, our simulations confirmed that formation of phosphoenolpyruvate from pyruvate via pyruvate orthophosphate dikinase neither needs to be active during the glucose simulations [12] nor in any other physiological state studied here. However, we found that the reverse reaction is active in all physiological states.

### Light and oxygen enhance the demethylation of DMSP

According to our results, light and the presence of oxygen stimulate the usage of the DMSP demethylation pathway. Interestingly, the effect depends on the carbon uptake rate. While oxygen always enhances the activity of the demethylation pathway, the effect of light is much more pronounced in physiological states with a medium uptake rate. On average, the cleavage pathway is used to a greater extend but the fraction of demethylation decreases as the uptake rate increases. This is consistent with measurements made using fifteen different *Roseobacter* strains [46]. Although DMSP may have become an abundant compound only recently [10], *D. shibae* and other *Roseobacters* seem to be well-suited to use it efficiently under various conditions. Whether the regulation of these pathways is really as sophisticated as predicted by our model remains an open question.

### Excess of reducing equivalents can cause aerobic denitrification

Our results support the hypothesis proposed in a recent review stating that under certain conditions, nitrate respiration is not only being used for energy generation but has some other function, which may be redox balancing [47]. As described above, the energy demand of the organism is more than covered by the aerobic anoxygenic photosynthesis under low nutrient conditions. Nevertheless, our simulations showed that a terminal electron acceptor is still required for optimal growth to be retained in the light. This is due to an excess of reducing equivalents produced by different metabolic processes. These reducing equivalents need to be reoxidized to retain redox homeostasis. Again, flux balance analysis is not able to exactly determine which terminal electron acceptor is preferably used to achieve this goal. However, we used flux variability analysis to identify alternate optimal solutions. Indeed, some solutions involve denitrification under aerobic illuminated conditions. Intriguingly, this behavior has been reported for *Roseobacter denitrificans* [48]. We speculate that aerobic denitrification might be the preferred way to dispose electrons *in vivo* because it does not waste carbon and produces multiple oxidized redox equivalents.

### Phosphofructokinase is probably of little use for *D. shibae*

*D. shibae* is very likely adapted to an oligotrophic marine environment and a regular day/night cycle [49]. Hence, the organism may benefit only moderately from phosphofructokinase activity. This is especially true during the day when the energy demand is covered by the aerobic anoxygenic photosynthesis in large parts. On the one hand, this suggests a permanent shutdown of the phosphofructokinase. On the other hand, another possible explanation is that light induces a down regulation of the phosphofructokinase in *D. shibae* because the energy generated in lower glycolysis is not needed. This would also explain the inactivity of the enzyme observed by Fürch et al. as their cultures were grown in constant light [12].

### Plasmid and single gene knock-out mutants

In general, it can be informative to simulate a great variety of physiological states for each knock-out mutant. Since some effects do not occur in all physiological states, more subtle differences between the mutants can be revealed this way. This would not be possible with the commonly used method, which relies on one or two minimal media states [50], [33].

The simulated loss of plasmids brought up two interesting aspects regarding the 153 kb and the 86 kb plasmid. The 153 kb plasmid harbors the only copy of a catalase (Dshi_3801), which decomposes hydrogen peroxide to oxygen and water. The reason for the decreased growth is that the hydrogen peroxide created during the production of pyridoxal 5′-phosphate and the operation of the glycine oxidase now have to be degraded by a cytochrome-c peroxidase. The reaction catalyzed by this enzyme depends on reducing equivalents and hence is metabolically more expensive. Albeit hydrogen peroxide is created by other metabolic reactions in our simulations, singlet oxygen is also created during aerobic anoxygenic photosynthesis, which imposes an elevated oxidative stress level on the organism [51]. Hence, the catalase gene might be a beneficial acquisition for *D. shibae*. Furthermore, this hypothesis is supported by the fact that the catalase gene is upregulated in response to light [11].

The 86 kb plasmid contains the complete synthesis pathway leading to dTDP-*α*-L-rhamnose, which is an important compound of the outer membrane. Hence, our simulations predicted no growth in case of a loss of this plasmid for an unmodified carbolipid content. However, pseudogenes and transposases located on the plasmid may indicate it is no longer needed by the organism [4]. An explanation might be that *D. shibae* is able to change its surface structure or biofilm formation capabilities.

### Shutdown of DMSP demethylation stimulates DMS production

For the first time, we demonstrated that in theory a single gene knock-out is sufficient to significantly enhance the production of the cloud-seeding molecule DMS. In case of the mutants with a blocked demethylation pathway, the DMSP must be degraded inevitable by the cleavage pathway to retain growth. Hence, the more than 60% increase of the DMS production can be explained by the fact that 39.0% of the DMSP degraded by the demethylation pathway (Figure 6) is now redirected to the DMS-producing cleavage pathway. A similar effect occurred when the gene coding for the L-serine ammonia-lyase was removed. Although the demethylation pathway was kept intact, its activity was greatly reduced. The reason is that the carbon atom bound to tetrahydrofolate during the demethylation of DMSP, can no longer be routed to the central carbon metabolism. This is due to the fact that the glycine hydroxymethyltransferase binds this carbon atom to glycine producing L-serine whose degradation to pyruvate and ammonia is now inoperative. Alternatively, a salvage via the methionine synthase would be possible but we found no methionine degradation pathway in *D. shibae*. As the growth on the other carbon sources is not affected, the mutants could be grown on succinate for example and transferred to DMSP for DMS production afterwards.

## Materials and Methods

### Microbial cultivation conditions and media

The *D. shibae* DFL12T wild-type strain [3] was inoculated in complex medium (40 g l^−1^ Marine Bouillon medium, Carl Roth, Karlsruhe, Germany) and incubated in darkness at 30°C and 150 rpm for 48 h before being diluted 1∶50 in freshly prepared defined artificial sea water medium (SWM) [12] containing the following components per liter of medium: 4.0 g NaSO_4_, 0.2 g KH_2_PO_4_, 0.25 g NH_4_Cl, 20.0 g NaCl, 3.0 g MgCl_2_°6 H_2_O, 0.5 g KCl and 0.15 g CaCl_2_·2 H_2_O, 0.19 g NaHCO_3_, 1 ml trace element solution (2.1 g Fe(SO_4_)⋅7 H_2_O, 13 ml 25% (v/v) HCl, 5.2 g Na_2_EDTA⋅2 H_2_O, 30 mg H_3_BO_3_, 0.1 g MnCl_2_⋅4 H_2_O, 0.19 g CoCl_2_⋅6 H_2_O, 2 mg CuCl_2_⋅2 H_2_O, 0.144 g ZnSO_4_⋅7 H_2_O and 36 mg Na_2_MoO_4_⋅2 H_2_O per liter) and 10 ml vitamin solution (0.2 g biotin, 2.0 g nicotinic acid and 0.8 g 4-aminobenzoic acid per liter). Succinate (2 to 4 g l^−1^) and glucose (3.6 g l^−1^) were used as sole carbon sources. Main cultures were inoculated from these overnight cultures in fresh artificial sea water medium (SWM) with an OD_600_ of 0.05.

### Determination of biomass composition

Main cultures of *D. shibae* DFL12T were cultivated in 300 ml baffled shaking flasks filled with 50 ml of SWM (2 gl^−1^ succinate as sole carbon source). Cells were harvested at an OD_600_ of 1 (mid-exponential phase). Quantifications of macromolecules (lipids, proteins, DNA, RNA) were performed at least in three independent experiments sampling triplicates. Average amounts of total macromolecules were calculated in relation to cell dry weight of *D. shibae* DFL12T.

#### Isolation and quantification of total lipids (after [52])

15 ml of cell suspension were transferred to 15-ml polypropylene tubes and pelleted (10,000 g, 4°C, 5 min). Pellets were resuspended in 1.5 ml 100% methanol. After addition of 5 ml 100% methyl-tert-butyl ether (MTBE) and mixing (1 h, room temperature) phase extraction was achieved by addition of 1.25 ml deionized H_2_O followed by vigorous mixing for 1 min and centrifugation (1,000 g, 20°C, 10 min). The organic phase was transferred into fresh tubes. The polar phase was reextracted with 2 ml of the solvent mixture MTBE/methanol/water (10∶3∶2.5, v/v/v). Combined organic phases were transferred into dried and weighed glas vials. After desiccation in a vacuum centrifuge (approximately 6 h) lipids were quantified gravimetrically.

#### Isolation and quantification of total proteins

5 ml of cell suspension were transferred to 15-ml polypropylene tubes and pelleted (10,000 g, 4°C, 5 min). After washing pellets with 0.1 M potassium phosphate buffer (pH 7.4), cells were resuspended in 1 ml 0.9% (w/v) NaCl. For cell lysis 1 ml 1.32 M NaOH was added to each sample, followed by an incubation in an ultrasonic bath at 80°C for 30 min. After cooling down, total protein quantification was performed according to [53] using the Folin&Ciocalteu's phenol reagent (Sigma-Aldrich GmbH, Steinheim, Germany).

#### Isolation and quantification of total genomic DNA

2 ml of cell suspension were pelleted (10,000 g, 4°C, 5 min) and cell walls disrupted by using the alkaline lysis method according to [54]. Pellets were resuspended in 700 *µ*l of a solution containing 50 mM glucose, 25 mM Tris/HCl (pH 8.0) and 10 mM EDTA (pH 8.0) and incubated for 10 min at room temperature. Extraction of genomic DNA was performed by adding a mixture of TE-buffer equilibrated phenol, chloroform and isoamyl alcohol (25∶24∶1). After centrifugation (13,000 rpm, room temperature, 2 min), the aqueous phase was transferred to a fresh tube. The phenol extraction step was repeated three times. In order to precipitate the DNA, 0.1 volumes of 3 M NaAc, pH 5.0, and an equal volume of 100% isopropanol were added to the aqueous phase and incubated overnight at 4°C, followed by centrifugation (13,000 rpm, room temperature, 15 min). A washing step with 70% (v/v) ethanol was done prior to drying of the DNA pellet. The dried DNA was resuspended in sterile water and quantified photometrically.

#### Isolation and quantification of total RNA

250 *µ*l of cell suspension was pelleted (10,000 g, 4°C, 5 min) and resuspended in 750 *µ*l TRIZol; (Invitrogen, Darmstadt, Germany). After incubation for 5 min at room temperature, 150 *µ*l chloroform were added to each sample. After vortexing for 15 s samples were centrifuged (12,000 g, 4°C, 15 min). Supernatant was transferred into a fresh RNase-free tube. RNA was precipitated by adding 375 *µ*l 100% isopropanol to the aqueous phase and centrifugation (12,000 g, 4°C, 10 min). A washing step with 75% (v/v) ethanol was done to remove excess salt from the pellet prior to drying the RNA pellet in a desiccator and resuspending in RNase-free water. RNA concentration was quantified photometrically.

#### Isolation and quantification of total polyhydroxybutanoate (PHB)

1 ml cell suspension was pelleted (10,000 g, 4°C, 5 min) and resuspended in 1 ml 2 N NaOH. After hydrolysis (30 min, 95°C) the sample was cooled down and neutralized with the equal amount of 2 N HCl. The sample was centrifuged (10,000 g, 4°C, 5 min) and the supernatant was transferred into a fresh reaction tube. A dilution series of the PHB standard (0.01…0.5 g/L) was prepared accordingly for calibration purposes. Measurements were performed on a La Chrome Elite HPLC equipped with an Aminex HPX-87H column according to [55].

### Phenotype MicroArray analysis

The experiments were carried out following the manufacturers' instructions (Biolog Inc., USA) with modifications. The inoculation and incubation solutions IF-0a GN/GP were adapted to the artificial sea water medium by adding a 10-fold concentrated SWM solution including vitamins and trace elements without carbon source (final concentration of components equal to SWM).

#### Growth condition

*D. shibae* DFL12T cells were taken from glycerol stocks and streaked out on MB-agar plates and incubated for 72 h at 30°C. A few colonies were taken one day before the Phenotype MicroArray Analysis and streaked out on MB agar and incubated overnight at 30°C.

#### Preparation of inoculation solution for microplates PM1 and PM2A

Cell suspensions were generated by resuspending colonies from MB-agar plates in 6 ml inoculation solution (5 ml 1.2× IF0a GN/GP (Biolog Inc., USA), 0.5 ml SWM (10-fold), 0.5 ml H_2_O) until a turbidity of 10% was reached. 2 ml of this inoculation solution were mixed with the incubation solution consisting of 8.33 ml IF0a GN/GP, 120 *µ*l DyeD (Biolog Inc., USA), 406 *µ*l H_2_O, 1 ml SWM (10-fold), 120 *µ*l vitamins, 24 *µ*l trace elements), resulting in a final turbidity of 60%. The microplates PM1 and PM2A (Biolog Inc., USA ) were inoculated with 100 *µ*l suspension per well and incubated for 72 h at 30°C in the Omnilog Reader (Biolog Inc., USA). Read-outs were automatically performed every 15 minutes and data stored for later analysis.

#### Data analysis

Data were imported into the Biolog software, background subtracted and the corresponding areas under the curve exported for each well. Further statistical analyses were performed in Excel.

### Carbon uptake rate

Cultivation of *D. shibae* DFL12T was performed as described above using 2 g l^−1^ succinate as sole carbon source. Sampling took place every hour until cultures (three in parallel) entered the early stationary growth phase (approximately 16 h). Sampling was performed by transferring 1 ml cell culture into 2-ml tubes. Cells were separated from the medium by centrifugation (10,000 g, 4°C, 5 min). Supernatants were transferred into fresh tubes and stored at −20°C until further processing with GC/MS.

#### GC/MS analysis

30*µ*l of the culture supernatant were mixed with 500 *µ*l deionized H_2_O containing 20 *µ*l ribitol (0.2 g l^−1^) prior to drying in a desiccator. A dilution series of the corresponding carbon source (0…60 *µ*g) was prepared accordingly for calibration purposes. Derivatization and measurement of samples was carried out according to [56] with the following exceptions: 40 *µ*l pyridine containing 20 mg ml^−1^ methoxyamine hydrochloride and 60 *µ*l N-methyl-N-trimethylsilyltrifluoroacetamide were used for derivatization; GC-MS analysis was performed on a Thermo DSQ II mass spectrometer (Thermo, San Jose, USA) with equal settings and the gas chromatograph was equipped with a Phenomenex Zebron ZB-5MS GuardianTM column (30 m×0.25 mm ID, 0.25 *µ*m film thickness). For data acquisition, the Xcalibur 1.2 software (Thermo Scientific) was used. All chromatograms were processed using MetaboliteDetector [57] for targeted analysis. The succinate concentrations of the supernatant samples were determined over the linear equation obtained by plotting the mean values of normalized peak areas of succinate of the calibration samples against the respective concentrations. The carbon uptake rate was calculated during maximum growth rate as a function of time and cell dry weight (*mmol/*(*gDW h*)).

### Determination of swimming velocities

For the comparison of bacterial motility *D. shibae* DFL12T was cultivated as described above using 2 g l^−1^ succinate as sole carbon source and 1.3 g l^−1^ glucose, respectively. During exponential growth phase subsamples were taken and transferred to a stage micrometer. To prevent false positive movements due to evaporation of medium the cover slip was sealed with nitrocellulose. Cells were monitored through a microscope (Zeiss Axiostar plus) equipped with a 40× objective. The bacterial movement was digitally recorded for 60 seconds with a Canon PowerShot A640 camera (640×480 pixels resolution, 16× zoom) which was connected to the microscope.

Five still images per second were extracted from the videos and the hqdn3d filter of FFmpeg version 0.8.5-6 was used to filter noise. Subsequently, all images were converted to grayscale and bright spots were mapped to black pixels with ImageMagick; version 6.7.7-10. Spot detection and tracking was performed using Icy version 1.3.1.0 [58]. Only tracks with a length of at least two were kept for further analysis.

### Aerobic anoxygenic photosynthesis

#### Cultivation

Continuous cultivation of *D. shibae* DFL12T was performed in 3 l minimal salt water medium with 2 g l^−1^ succinate in a 3.6-l double jacket glass chemostat (Labfors4, INFORS HT, Einsbach, Germany) equipped with a LED-illumination unit (photon flow 130 *µ*E m^−2^s^−1^) at 30°C, pH 8.0, with an aeration of 3l min^−1^ and a constant stirring speed of 250 rpm. The pH was adjusted automatically with 0.5 M H_2_SO_4_ and 0.2 M NaOH. The off-gas was analyzed on-line using O_2_ and CO_2_ gas sensors (BlueSens gas sensor GmbH, Herten, Germany). To avoid disturbance of the experiment by external light, the chemostat was covered with aluminum foil prior to inoculating with a SWM overnight-culture to an OD_600_ of 0.05. After 13 h of batch cultivation the oxygen saturation of the culture reached the minimum and the continuous cultivation was started using a dilution rate of 0.1 h^−1^. Light shift experiments were started at steady state of the culture, when constant levels of the oxygen saturation of the medium and constant off-gas CO_2_ and O_2_ concentrations were reached (after approx. 6 residence times).

#### Metabolome analysis

Samples for intracellular metabolome analysis were taken from the chemostat via a sterile sampling port after 1 h of illumination within two consecutive day and night cycles (12 h/12 h). For metabolite extraction, cellsuspensions were pelleted at 10,000 g, 4°C for 3 minutes (centrifuge 5810 R, Eppendorf, Germany). Supernatants were discarded and cell pellets resuspended in 20 ml pre-cooled 3.5% NaCl (w/v) before further centrifugation. The washing step was repeated once. The resulting cell pellets were resupended in 0.75 ml cold methanol containing 15 *µ*l ribitol (0.2 g l^−1^) as internal standard. Cell lysis was achieved by ultrasonification (15 min, 70°C). After cooling down on ice for 2 min, the metabolite extraction was carried out by adding 0.75 ml deionized H_2_O, vortexing for 1 min and adding 1 ml chloroform to each sample. After a subsequent centrifugation (10,000 g, 4°C, 5 min) 1 ml of the polar phases were subjected to a centrifugal-vacuum concentrator to allow evaporation. The two-step derivatization reaction of the dried samples, the GC-MS analyses with a Leco Pegasus 4D GC×GC-TOF-MS and the data analysis were performed as described by Zech et al. [59].

### Metabolic network reconstruction

The starting point of our reconstruction process was the genome annotation of *Dinoroseobacter shibae* DFL12T provided by the EnzymeDetector database [25]. This database aggregates annotations from different sources and assigns a relevance score to each entry indicating the level of confidence. To exclude poorly annotated enzymes, we selected only entries with a minimum relevance score of 9. Occasionally, multiple enzymes are annotated for one open reading frame. In such cases the two best entries were compared with each other. Both were kept if their score was equal or above 13. Otherwise only the entry with the best score was kept. Furthermore, manual additions were made to the annotation during the reconstruction to fill gaps in the metabolic network. The final annotation used for the creation of the model can be found in the Supplementary Dataset S1.

To create the metabolic model, the enzymes from the annotation were mapped to the corresponding chemical reactions via their EC number. This mapping was based on the MetaCyc database version 16.0 [60]. Next, spontaneous reactions were added under the condition that all educts of the reaction were already part of the model. This step has been repeated until no new reactions were found. Transport and boundary reactions were added manually to allow certain nutrients, additional vitamins and waste products to enter or leave the system. This preliminary model was iteratively refined by adding additional enzymes and reactions to fully reproduce growth under different conditions. Stoichiometric balancing was performed computationally for all reactions. The non-growth-associated maintenance requirement (nGAM) and the growth-associated maintenance requirement (GAM) were assumed to be the same as in *E. coli* (3.15 *mmol* ATP/(g*_DW_ h*) and 53.95 *mmol* ATP/(g*_DW_ h*) [33]. While the first value models the energy demand of processes not related to growth like DNA repair and preservation of turgor pressure, the second value accounts for the energy needed for reproduction. Most of this energy is needed for the synthesis of proteins, DNA, and RNA. The number of protons in *mmol/*(*g_DW_ h*) needed to drive the flagellar motor *P_motility_* has been estimated based on the number of protons needed for one rotation of the motor *N* = 1200 [61], the average number of rotations per second *v* = 10 *s* ^−1^ [62], and the average dry weight of one *Roseobacter* cell *m* = 300*fg* [63]:
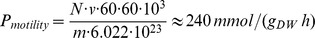
However, only about 10% of the organisms in a culture of marine bacteria are motile during the early exponential growth phase modeled here [24]. Hence, we constrained the motility proton flux to 24 *mmol/*(*g_DW_ h*).

### Computational analysis

We simulated the physiological states using flux balance analysis [64], [65]. Furthermore, each flux was tested for variability under the additional constraint of optimal biomass production by (fast) flux variability analysis [66], [67]. All computational analyses were carried out on a computer equipped with a 2.67 GHz Intel Core i7 CPU and 4 GB of RAM. The software in use was the metano toolbox (Riemer *et al*, in preparation, http://metano.tu-bs.de). Altogether, the simulations took about four days to finish. For further evaluation, the resulting fluxes were stored in a relational database. Single gene knock-outs were simulated by constraining the flux through all reactions associated with that particular gene to zero.

## Supporting Information

Dataset S1 Complete list of all genes covered in *i*Dsh827.
(XLS)

Dataset S2 Final metabolic model in SBML format (zipped).
(ZIP)

Figure S1 Graphical representation of the distribution of evidence scores.
(TIF)
