# Supplementary figures and images for: Swimming in Light: A Large-Scale Computational Analysis of the Metabolism of Dinoroseobacter shibae
Source: PLoS Comput Biol. 2013 Oct 3;9(10):e1003224. doi: 10.1371/journal.pcbi.1003224 (PMC3789786; doi:10.1371/journal.pcbi.1003224)

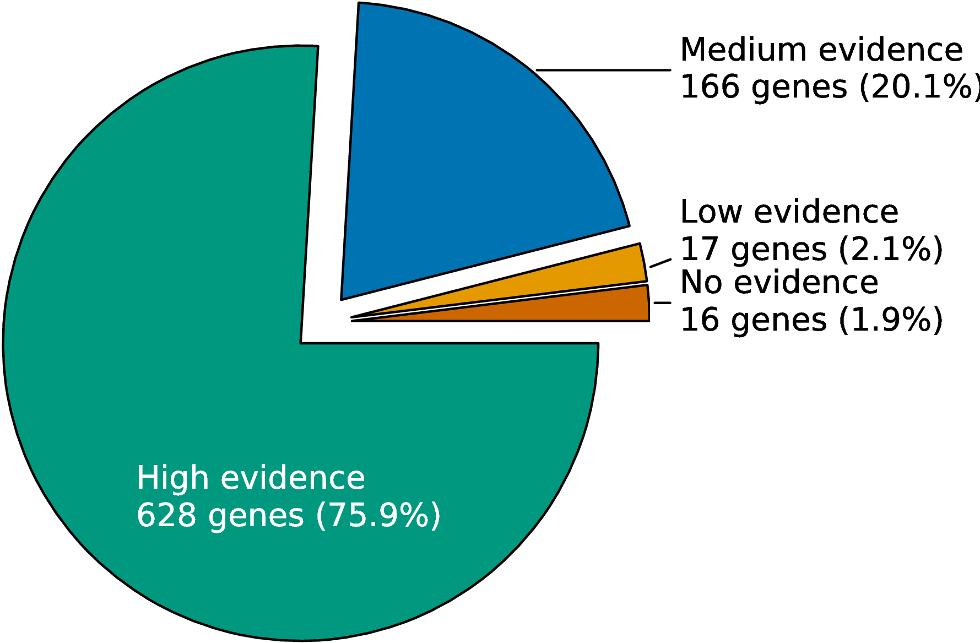

Supplement: Figure S1 — Graphical representation of the distribution of evidence scores. (TIF) [file pcbi.1003224.s003.tif]
